# Gut microbiota differences in stunted and normal-lenght children aged 36–45 months in East Nusa Tenggara, Indonesia

**DOI:** 10.1371/journal.pone.0299349

**Published:** 2024-03-29

**Authors:** Ingrid S. Surono, Ilia Popov, Sanne Verbruggen, Jessica Verhoeven, Pratiwi D. Kusumo, Koen Venema

**Affiliations:** 1 Faculty of Engineering, Food Technology Department, Bina Nusantara University, Jakarta, Indonesia; 2 Centre for Healthy Eating & Food Innovation, Maastricht University—Campus Venlo, Venlo, The Netherlands; 3 Faculty of Medicine, Universitas Kristen Indonesia, Jakarta, Indonesia; Washington State University - Spokane, UNITED STATES

## Abstract

The role of the gut microbiota in energy metabolism of the host has been established, both in overweight/obesity, as well as in undernutrition/stunting. Dysbiosis of the gut microbiota may predispose to stunting. The aim of this study was to compare the gut microbiota composition of stunted Indonesian children and non-stunted children between 36 and 45 months from two sites on the East Nusa Tenggara (ENT) islands. Fecal samples were collected from 100 stunted children and 100 non-stunted children in Kupang and North Kodi. The gut microbiota composition was determined by sequencing amplicons of the V3-V4 region of the 16S rRNA gene. Moreover, fecal SCFA concentrations were analyzed. The microbiota composition was correlated to anthropometric parameters and fecal metabolites. The phyla Bacteroidetes (Bacteroidota; *q* = 0.014) and Cyanobacteria (*q* = 0.049) were significantly higher in stunted children. Three taxa at genus levels were consistently significantly higher in stunted children at both sampling sites, namely *Lachnoclostridium*, *Faecalibacterium* and *Veillonella* (*q* < 7 * 10^−4^). These and 9 other taxa positively correlated to the z-score length-for-age (zlen), while 11 taxa negatively correlated with zlen. Several taxa also correlated with sanitary parameters, some of which were also significantly different between the two groups. All three fecal SCFA concentrations (acetate, propionate and butyrate) and their total were lower in stunted children compared to non-stunted children, although not significant for butyrate, indicating lower energy-extraction by the gut microbiota. Also, since SCFA have been shown to be involved in gut barrier function, barrier integrity may be affected in the stunted children. It remains to be seen if the three taxa are involved in stunting, or are changed due to e.g. differences in diet, hygiene status, or other factors. The observed differences in this study do not agree with our previous observations in children on Java, Indonesia. There are differences in infrastructure facilities such as clean water and sanitation on ENT and Java, which may contribute to the differences observed. The role of the gut microbiota in stunting therefore requires more in depth studies.

**Trial registration**: the trial was registered at ClinicalTrials.gov with identifier number NCT05119218.

## Introduction

Stunting in children is defined as those children being too short for their age, defined as a stature with a height-for-age (or length-for-age as used by the World Health Organization [WHO]) score more than 2 standard deviations below the reference standard [1]. This is mostly due to poor nutritional intake [2], but also hygiene practices, including access to clean water, together with socioeconomic influences, all contribute to the health status of these children and influences growth. Stunting has become a major health problem in the world. Because a mother, who experienced stunting in childhood, will likely bear a stunted child, this is a cyclic process [3]. The linear growth retardation in these children already begins in utero and continues into infancy and early childhood [4]. The 2020 data from UNICEF/WHO/World Bank Group [5] reveals that more than one-fifth of children under five-years-old, a staggering 144 million children worldwide, were stunted due to chronic malnutrition. Of these 54.3% were found in Asia, and 39.9% in Africa. This stunting was largely irreversible after the child’s second birthday.

Stunting therefore has been identified by UNICEF as a major global health priority [6]. In line with that, the WHO has set a target to reduce stunting by 40% between 2010 and 2025. Moreover, improving the identification, measurement and understanding of stunting is part of recommended actions by WHO in scaling up the prevention [7]. Although frequently stunting is equated with malnutrition and chronic infection, the latter mostly due to lack of hygiene, there is accumulating evidence that the link with malnutrition and infection alone may not be entirely valid, as evidenced in a population of Indonesian children [8]. This study “… seriously questions the concept of stunting as prima facie evidence of malnutrition and chronic infection” [8].

Indonesia is considered to have a high prevalence of stunting overall. According to the global nutrition report [9], Indonesia experienced a slight decrease in stunting from 42.4% in 2001 to 36.4% in 2013. Based on current data, the cases of stunting have been decreasing further to 30.8% in 2018 [10] and to 27.7% in 2019 [9], but still amounts to 4.6 million children in 2019. Although there is a trend for a gradual decrease, the burden of stunting in Indonesia is still above the rate of stunted children in the Southeast Asia region (24.7%) [9], and depends heavily on the district within Indonesia in which these children live [11]. Around 53% of the population now live in urban areas, whereas the remainder live in rural areas spread over the many islands, with large differences in hygiene practices and facilities [5].

The gut microbiota has close links to food digestion, food absorption and intestinal function. Persistent undernutrition in childhood will change the (development of the) healthy composition of the gut microbiota (referred to as dysbiosis) [12]. Vice versa, gut microbiota dysbiosis has been associated with malnutrition and reduced plasma essential amino acid levels in children from two low-income countries: Malawi and India [13]. There is accumulating evidence that the gut microbiota influences body weight regulation, both in overweight and obesity [14] and well as in anorexia nervosa [15]. The microbial activity, in terms of metabolites produced by the gut microbiota, has been shown to play a role in weight regulation, with a particular role for the short-chain fatty acids (SCFA; primarily acetate, propionate and butyrate) [16,17]. Also in stunting, an altered gut microbiota is linked to the pathophysiology of stunting, where such alteration have even been detected prior to actually observing delayed growth in children between 6 and 23 months [18]. In the context of stunting, is has been observed that Enterobacteriaceae (of the Proteobacteria phylum [or Pseudomonadota phylum as it has been renamed to [19]], which is often associated with human pathogenicity) are increased in concordance with impaired digestion/absorption and localized gut inflammation [20,21].

Gut microbiota differences are attributable to our own genetics, diet and age, amongst many other variables. The composition is individual and region specific, with different compositions in Asia, Europe, US and Africa [22–25], partly correlated to differences in diet for Asian children [22], although that data is obtained in children at two location in 5 countries (Bali and Yogyakarta for Indonesia) and may not be representative for the whole country, or rural areas. In the Asian Microbiome Project, particular profiles in school-aged children could be linked to intake of resistant starch (e.g. from certain rice varieties) and chicken [22].

The profile of fecal microbiota of apparently healthy and those of stunted Indonesian children aged 3–5 years needs to be explored to find out how gut microbial community structure changes with nutritional status. In our earlier study in 2019 on microbiota in stunted children on Java, Indonesia [26,27], we found that the composition was correlated to nutritional status. In stunted children, the relative abundance of Bacteroidetes was significantly lower than in normal children, while Firmicutes was significantly higher. At the genus level, 14 taxa were significantly different between groups. Overall “*Prevotella 9*” was the most abundant genus (average of 27%), and it was significantly lower in stunted children than in normal children (23.5% vs. 30.5%, respectively). The two districts on Java that were chosen then (Pandeglang, Banten province and Sumedang, West Java province), were amongst the regions in Indonesia with had the highest prevalence of stunting at 22% and 37.8%, respectively [8], despite the fact that both districts are close to capital cities in Indonesia. Based on the Study on the Nutritional Status of Indonesia (2021) [11] East Nusa Tenggara has 15 districts with high stunting prevalence; among others, Kupang city and Southwest Sumba (26.1% and 44%, respectively).

The aim of the study was to explore the interrelationships between the gut microbiota profile and the nutritional status of children in East Nusa Tenggara (Kupang–urban versus North Kodi–rural), and to identify key microbial taxa associated with stunting. This information can subsequently be used to correct the observed dysbiosis in these stunted children by optimal interventions to manage malnutrition, through proper dietary interventions, and access to proper clean water, personal hygiene and sanitation practices, which in turn modulate the gut microbiota.

## Materials & methods

### Study design

A cross-sectional study was conducted in children aged 3–5 years old with stunting (*n* = 100) and those without stunting (*n* = 100), at two locations: Kupang and North Kodi, in the East Nusa Tenggara (ENT) Province, Indonesia from November 06, 2021 to March 08, 2022.

The nutritional status of each child included in this study was quantified using the WHO recommended three nutritional Z-scores namely, (height or) length-for-age (zlen); weight-for-age (zwei) and weight-for-length (zwfl), where in addition the Z-score for bmi-for-age (zbmi) was calculated. In addition, age and anthropometric measurements (height (or length), weight) based on Department of Health Ministry of Indonesia Regulation and WHO were recorded. For stunting, the thresholds for zlen are: ‘severely stunted’ (<-3 SD); ‘stunted’ (-3 SD to < -2 SD); ‘normal’ (-2 SD to +3 SD); ‘tall’ (> +3 SD). Furthermore, in order to obtain an overall measure of the nutritional status of these children, the children were classified in zwei categories: ‘severely wasted’ (<-3 SD); ‘wasted’ (-3 SD to < -2 SD); ‘normal’ (-2 SD to +1 SD); ‘possible risk of overweight’ (+1 SD to +2 SD); ‘over weight’ (> +2 SD to +3 SD); ‘obese’ (> +3 SD) [1]. Several sanitary variables were scored as well. Those variables that were significantly different or correlated to microbial taxa included: material of the ceiling of accommodation, material of the walls, material of the floor (defined as: 0: earthen, 1: panel board/bamboo/plaster cement, 2: tiles), ventilation in kitchen (defined as: 0: none, 1: <10% of floor area, 2: >10% of floor area), ventilation in living room (defined as: 0: none, 1: <10% of floor area, 2: >10% of floor area), clean water facilities, waste water facilities, sanitary facilities (defined as: 0: no toilet, 1: hole in the ground without cover, 2: hole in the ground with cover, 3: septic tank, 4: complete toilet), toilet (defined as: 1: public or 2: private), toilet location (defined as: 1: ‘around’ or 2: inside), handwashing after toilet visit (defined as: 1: no or 2: yes), handwashing with flowing water (1: no or 2: yes), handwashing with soap (defined as: 1: no or 2: yes).

This was a follow-up of a previous study on Java, which also include 100 stunted and 100 non-stunted children [27]. The same conditions (such as sample storage, primers, PCR cycles, sequencing protocol, bioinformatic tools, etc.) were used in this study as the one performed by the same team on Java earlier.

### Collection of feces

Fecal samples were collected on site from stunted children and non-stunted children and immediately placed in a cooler with ice-packs, and shipped the same day to the lab in Jakarta on dry ice. In the lab, 0.5 g of the feces was mixed with 4.5 ml of Zymo buffer (Baseclear, Leiden, the Netherlands) and kept at room-temperature prior to extraction of DNA. Another 0.5 gram was weighed in a tube for extraction of SCFAs.

### DNA isolation and sequencing of the V3-V4 region of the 16S rRNA gene

DNA isolation and sequencing of barcoded amplicons of the V3-V4 region of the 16S rRNA gene were essentially performed as described before [28] according to established protocols provided by Illumina (Illumina, Eindhoven, the Netherlands). The sequencing was carried out using the Illumina MiSeq system (San Diego, CA, USA). Sequences were converted into FASTQ files using BCL2FASTQ pipeline, primer sequences were removed and quality trimming was applied based on the Phred quality score. QIIME 2 software was used for microbial analyses [29]. The sequences were classified using SILVA (version 132) as a reference 16S rRNA gene database. Alpha- and β-diversity metrics were obtained using QIIME2 as well. The Amplified Sequence Variants (ASVs) table was filtered for those taxa that occurred in more than 20% of the total number of fecal samples. Blanks, positive controls (a fecal sample that is tested with each sequence run) and a mock sample (also tested with each sequence run) were taken along and behaved as expected (i.e.: no reads, the expected composition at 10.000 reads, and the expected relative abundance at 10.000 reads, respectively).

### Short chain fatty acid analyses

SCFAs were analyzed by GC-MS, essentially as described before [28]. Briefly, fecal samples were mixed 1:1 (w:w) with PBS, vortexed for 2 minutes and centrifuged at 14,000×g for 10 min. One hundred and fifty μl of the supernatant were mixed with 550 μl internal standard solution, containing methanol, internal standard (2 mg/ml 2-ethyl butyric acid), and formic acid (20%). The analysis was carried out on a GC-MS (8890 GC System; Agilent Technolgies, Amstelveen, the Netherlands) equipped with a PAL3 RSI 85 autosampler (Agilent) by injecting 1 μl sample on a DB-FATWAX Ultra Inert column (30 m, 0.25 mm, 0.25 μm, Agilent). The temperature settings of the injector port, oven, flame-ionization detector and mass spectrometer detector were 250, 200, 275 and 225°C, respectively. The flow rate over the column was 1.2 ml|/min. The MS Quantitative Analysis (Quant-My-Way) software from Agilent was used to determine concentrations of SCFA. LODs were: 0.95 mmol/L for acetate and 0.91 mmol/L for both propionate and butyrate. The lowest concentrations found in the samples were at least 2-fold higher than the LOD.

### Statistical analyses

Correlations between ASVs and different categorical variables, such as stunted/non-stunted, gender, sampling site, or sanitary variables were investigated using the non-parametric Kruskal Wallis test corrected for multiple comparisons by Benjamin-Hochberg false discovery rate (FDR), by using the software package R (3.5.3) (R Core Team, http://www.R-project.org/) in RStudio. Non-parametric Spearman’s rank-order correlations were obtained between ASVs and continuous variables, such as age, height and weight, and the concentrations of SCFAs present in feces. *Q*-values (adjusted *p*-values after FDR) were considered significantly different at a strict cut-off of *q* < 0.05. The map of Indonesia with the taxa plotted in pie-charts was made with the R-package ‘maps’ (https://cran.r-project.org/web/packages/maps/index.html). Beta-diversity was visualized using principal coordinate analysis (PCoA) plots in QIIME2. Permutational multivariate analysis of variance (PERMANOVA; [30]) was performed to test the significance of beta-diversity (weighted and unweighted UniFrac, Bray-Curtis dissimilarity and Jaccard similarity) between normal and stunted children in QIIME2.

### Ethics approval and consent to participate

The study protocol was approved by the Ethics Committee of Research Institute of YARSI University and registered at ClinicalTrials.gov with identifier number NCT05119218. Parents or children’s caregivers received oral and written information and signed a letter of consent before children included in the study.

## Results and discussion

### Characteristics of the children 36–45 months of age

**Fig 1:**
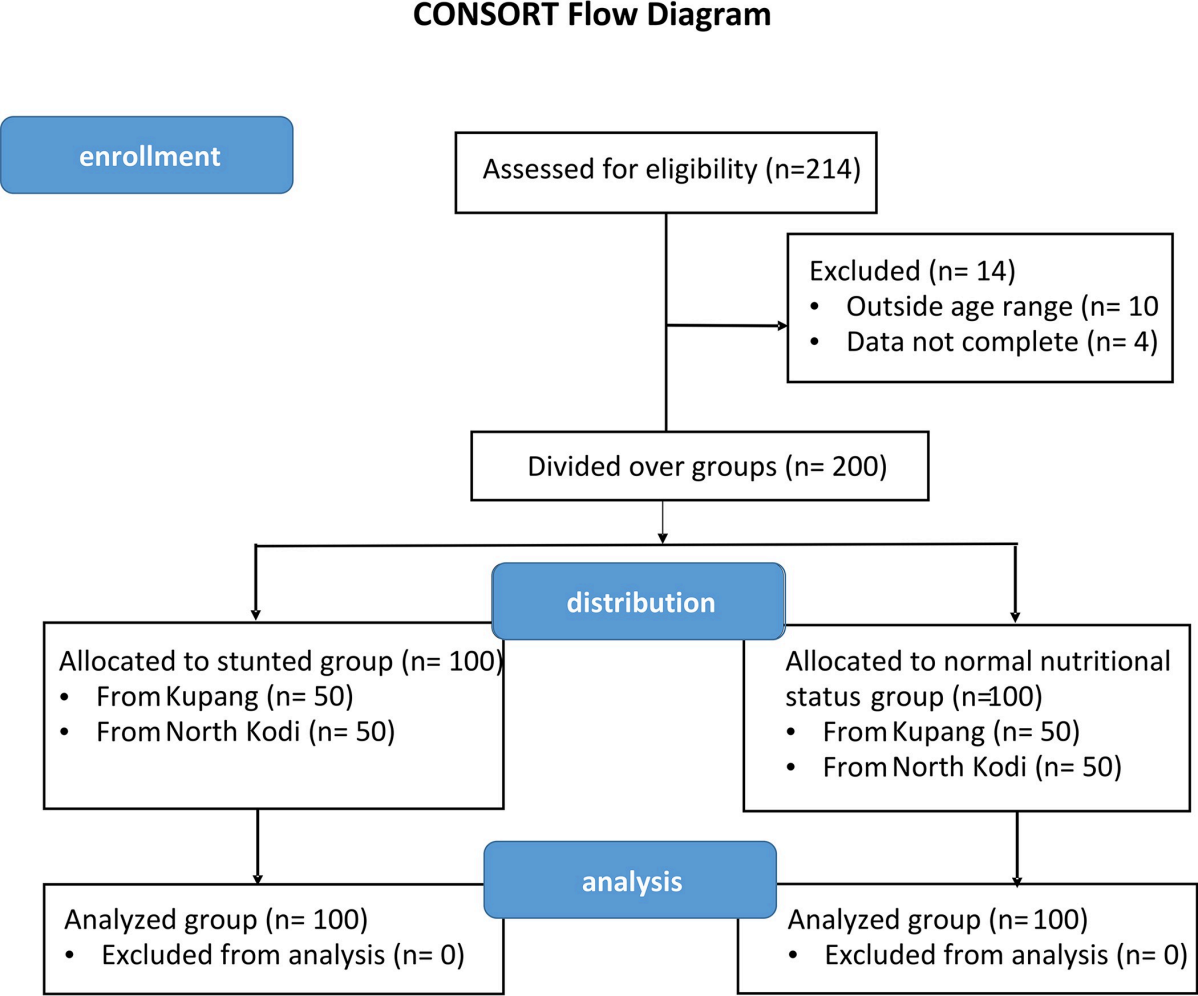
CONSORT flow diagram of the cross-sectional study in East Nusa Tenggara, Indonesia.

This was a cross-sectional study, without intervention. Fig 1 shows the CONSORT flow chart of the study. Table 1 shows the anthropometric parameters of the two groups of children segregated based on their zlen. Stunted children were on average 8 cm (9%) shorter compared to the children with normal zlen (Table 1, *p* = 1.8 * 10^−10^). They were also significantly lighter in bodyweight (*p* = 1.5 * 10^−6^), as well as different in BMI (*p* = 2.6 * 10^−6^). The age of the two groups did not significantly differ (*p* = 0.62).

**Table 1:** Anthropometrics of the groups.

	normal	stunted	*p*-value
Gender (M/F)	43/57	47/53	
Age (month; mean ± SD)	39.74 ± 3.27	39.68 ± 3.33	0.62
Weight (kg; mean ± SD)	12.90 ± 1.39	11.72 ± 1.19	1.5 * 10^−6^
Height/length (cm; mean ± SD)	94.43 ± 4.42	86.44 ± 3.45	1.8 * 10^−10^
BMI (kg*m^-2^; mean ± SD)	14.52 ± 1.44	15.78 ± 2.11	2.6 * 10^−6^

**Fig 2:**
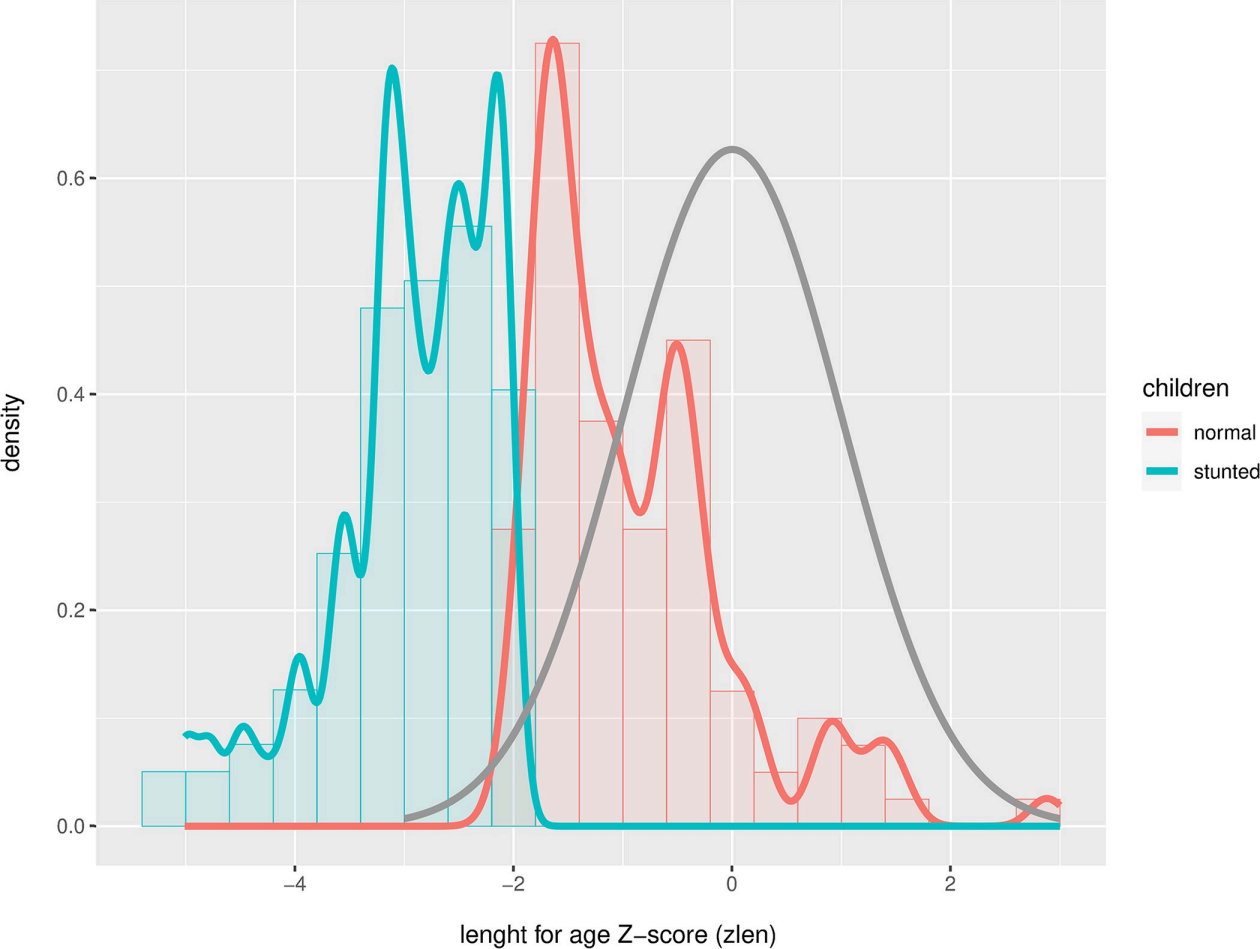
Length for age Z-score of the Indonesian children (stunted: Green; normal: Red) plotted against the WHO reference dataset (grey).

Fig 2 shows the distribution of the zlen of non-stunted children (green) and stunted children (red), plotted against the WHO reference dataset (grey). Stunting is defined as Z-score < -2, and severe stunting as Z-score < -3. Some of the children had a Z-score lower than -4 (*n* = 8), with the lowest score being -5.01. From the distribution of the children considered non-stunted, it is clear that most of these (*n* = 86 of 100) are to the left of the zero-line in the WHO reference data-set, and therefore on average are still shorter than the average length-for-age in the WHO-dataset. This was similar to our previous study on stunted children in comparison to non-stunted children on Java, Indonesia [27].

### Differences in sanitary parameters between the groups

Several sanitary parameters were significantly different between stunted and non-stunted children (Table 2). The remainder of the parameters did not reach significance.

**Table 2:** Sanitary parameters that were significantly different between the groups†.

	stunted	normal	*p*-value
floor material	0.80 ± 0.67	1.00 ± 0.72	0.041 *
kitchen ventilation	1.27 ± 0.93	1.00 ± 0.93	0.033 *
living room ventilation	0.82 ± 0.69	1.00 ± 0.64	0.045 *
public toilet	0.86 ± 0.29	1.00 ± 0.25	0.0002 *
location of (public) toilet	0.93 ± 0.25	1.00 ± 0.23	0.044 *
hand washing after toilet	0.96 ± 0.17	1.00 ± 0.11	0.047 *
hand washing with flowing water	0.90 ± 0.32	1.00 ± 0.34	0.033 *
handwashing with soap and/or water	0.94 ± 0.19	1.00 ± 0.18	0.024*

† floor material (0; earthen, 1: Panel board/bamboo/plaster cement, 2: Tiles), kitchen ventilation (0: None, 1: <10% of floor area, 2: >10% of floor area), living room ventilation (0: None, 1: <10% of floor area, 2: >10% of floor area), public toilet (1: Public or 2: Private), toilet location (1: Public,‘around’, 2: Public, inside, 3: Private, ‘around’, or 4: Private, inside), handwashing after toilet visit (1: No or 2: Yes), handwashing with flowing water (1: No or 2: Yes), handwashing with soap and/or(flowing) water (1: Neither, 2: Flowing water, 3: Water and soap, or 4: Flowing water and soap). Arbitrary values of the non-stunted group (normal) were set to 1, and values for the stunted group were subsequently normalized to the normal group.

On all hygienic parameters in Table 2, except kitchen ventilation, the stunted group scored lower, meaning worse, than the normal group. This has been observed before in Indonesia [31,32], and elsewhere [33] before, reflecting not only nutritional intake, but also hygiene factors that are responsible for stunting.

### Gut microbiota differences between stunted and non-stunted children

The gut microbiota of the stunted children (S) from Kupang and North Kodi were compared to those of the non-stunted children (N) from the same region. Kupang is the capital city and the administrative center of East Nusa Tenggara. It is the largest city in East Nusa Tenggara and an urban area with the population of approximately 443,000 people. It is categorized as a semi-arid area due to relatively low rainfall, tropical, and dry climate which also tends to be influenced by the wind. North Kodi on the other hand is a rural area consisting of mountains and hills with a long dry season. The North Kodi community lives relatively far from urban areas.

As observed by many researchers before, the major phyla were Firmicutes (the recent new phyla name being Bacillota [19]) and Bacteroidetes (Bacteroidota). This was followed by Proteobacteria (Pseudomonadota). Both Actinobacteria (Actinomycetota) and Verrucomicrobia (Verrucomicrobiota) were present at low prevalence. Sixteen other phyla were present only in some samples. After filtering for those that were present in at least 20% of the samples, 10 phyla remained (Table 3). Of these Bacteroidetes were significantly higher in stunted children (23.9% ± 10.8% vs 20.0% ± 11.1%). Also Cyanobacteria (with overall very low abundance and prevalence) were significantly higher in stunted children (0.32% ± 0.70% vs. 0.17% ± 0.28%).

**Table 3:** Gut microbiota composition at the phylum level (mean ± SD)†.

Phylum	N	S	*p*-value
Euryarchaeota (Methanobacteriota)	0.12% ± 0.43%	0.13% ± 0.27%	0.81
Actinobacteria (Actinomycetota)	4.53% ± 5.19%	3.54% ± 4.07%	0.14
Bacteroidetes (Bacteroidota)	20.03% ± 11.06%	23.89% ± 10.84%	0.014 *
Cyanobacteria	0.12% ± 0.43%	0.13% ± 0.27%	0.048 *
Firmicutes (Bacillota)	60.19% ± 13.98%	58.19% ± 12.63%	0.29
Fusobacteria (Fusobacteriota)	0.08% ± 0.28%	0.06% ± 0.20%	0.74
Proteobacteria (Pseudomonadota)	12.82% ± 13.88%	11.66% ± 11.19%	0.52
Saccharibacteria	0.10% ± 0.42%	0.04% ± 0.09%	0.17
Tenericutes (Mollicutaeota)	0.32% ± 0.99%	0.71% ± 3.05%	0.24
Verrucomicrobia (Verrucomicrobiota)	1.49% ± 4.35%	1.17% ± 2.92%	0.54

† Phyla have been named according to the old nomenclature, with new nomenclature in brackets for those that we could find the new nomenclature, accepted [19] or proposed [34]; N = normal, S = stunted

*: *p* < 0.05.

With respect to α-diversity, the Shannon- and Faith’s phylogenetic diversity (PD-)indices were different between stunted children and non-stunted children (*q* = 0.084 and 0.0072, respectively). Evenness (*q* = 0.245) and observed_features (*q* = 0.056; which is called observed_OTUs in QIIME2, but measures ASVs) were not significantly different (Table 4), although the latter showed a trend to be higher in stunted children. The α-diversity was also compared between the urban and rural sampling sites, and evenness was significantly different between sampling sites (Table 4). This led us to split up the samples and compare stunted *versus* normal per sampling site. Like for the complete dataset, for samples collected in Kupang, the Shannon- and Faith’s PD-indices were different between stunted children and non-stunted children (Table 4).

**Table 4:** *P*-values for alpha and beta-diversity metrics†.

** **	** **	** **	**α-diversity**				** **	**β-diversity**			
*East Nusa Tenggara*			*Shannon*	*Faith’s PD*	*evenness*	*observed features*	* *	*unweighted UniFrac*	*weighted UniFrac*	*Bray-Curtis*	*Jaccard*
stunted	stunted	normal	**0.0084**	**0.0072**	0.245	0.056		**0.0010**	**0.0070**	**0.0010**	**0.0010**
site	Kupang	North Kodi	0.962	0.505	**0.030**	0.137		**0.0010**	**0.0010**	**0.0010**	**0.0010**
combined site & stunted	Kupang normal	Kupang stunted	**0.0019**	**0.0002**	0.154	0.315		**0.0012**	**0.0012**	**0.0012**	**0.0012**
	Kupang normal	North Kodi normal	0.0761	0.098	0.082	**0.0012**		**0.0012**	**0.0012**	**0.0012**	**0.0012**
	Kupang normal	North Kodi stunted	0.352	0.223	0.082	0.982		**0.0012**	**0.0012**	**0.0012**	**0.0012**
	Kupang stunted	North Kodi normal	0.352	0.056	0.556	**0.039**		**0.0012**	**0.0012**	**0.0012**	**0.0012**
	Kupang stunted	North Kodi stunted	**0.050**	**0.0076**	0.580	**0.0054**		**0.0012**	**0.0012**	**0.0012**	**0.0012**
	North Kodi normal	North Kodi stunted	0.352	0.569	0.920	0.314		0.086	**0.034**	**0.012**	**0.020**
											
*Java + East Nusa Tenggara*							* *				* *
stunted	stunted	normal	**0.0028**	**0.0033**	**0.0018**	**0.020**		**0.0010**	**0.048**	**0.0020**	**0.0010**
province	East Nusa Tenggara (ENT)	Java	**0.024**	0.148	0.983	**0.0060**		**0.0010**	**0.0010**	**0.0010**	**0.0010**
combined province & stunted	ENT normal	ENT stunted	0.246	0.108	0.084	0.251		**0.0012**	**0.0036**	**0.0012**	**0.0012**
	ENT normal	Java normal	0.721	0.762	0.520	0.251		**0.0012**	**0.0015**	**0.0012**	**0.0012**
	ENT normal	Java stunted	**0.0032**	**0.031**	0.073	**0.0055**		**0.0012**	**0.0015**	**0.0012**	**0.0012**
	ENT stunted	Java normal	0.422	0.267	0.058	0.879		**0.0012**	**0.0015**	**0.0012**	**0.0012**
	ENT stunted	Java stunted	**0.018**	0.194	0.801	0.0510		**0.0012**	**0.0015**	**0.0012**	**0.0012**
	Java normal	Java stunted	**0.015**	0.068	**0.028**	0.0996		**0.015**	0.052	**0.008**	**0.029**

† significance was determined using Kruskal-Wallis (alpha-diversity) or PERMANOVA (beta-diversity), with pairwise Kruskal-Wallis when there were more than 2 group (i.e. for ‘combined site & stunted’ and ‘combined province & stunted’). Data from the Java study is from [27].

However, none of the α-diversity-indices were significantly different for samples collected in North Kodi. Moreover, there were significant differences in Shannon-, Faith’s PD- and observed_features-indices between samples collected from stunted children in Kupang compared to those of children in North Kodi. Observed_features was also different between samples from non-stunted children from Kupang and North Kodi (Table 4).

**Fig 3:**
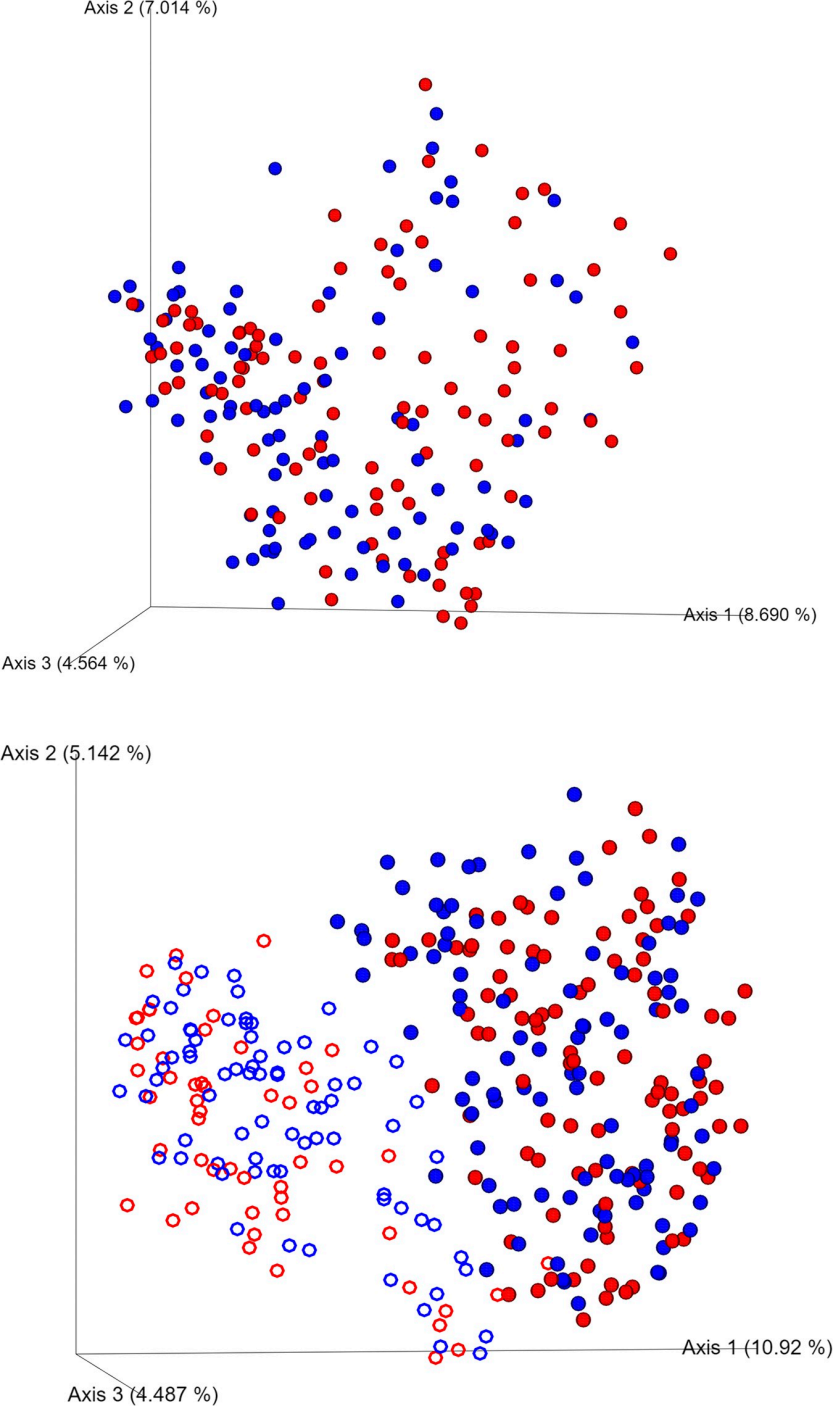
Principal coordinate analysis plot of Bray Curtis dissimilarity for A) non-stunted (red) and stunted (blue) children of the current study in East Nusa Tenggara, B) the non-stunted (red) and stunted (blue) children of the current study in East Nusa Tenggara (full circles) and our previous study on Java (open circles).

With respect to β-diversity, except for the unweighted UniFrac between samples from stunted and non-stunted children in North Kodi, all other metrics were significant, indicating that there were not only differences between children being stunted or not, but also between collection sites and the interaction between these two (Table 4). Fig 3A shows the principal coordinate analysis (PCoA) of the Bray-Curtis dissimilarity between the samples, colored by being stunted or not. The PCoA plot for the other indices are shown in S1 Fig.

Given the differences observed between the two sampling sites (urban and rural), we wondered whether the Kupang children might cluster closer to the samples we collected earlier in two cities on the western part of Java (Pandeglang and Sumedang, in Banten and West Java provinces, respectively) [27]. The β-diversity comparisons show significant differences between these two studies (Table 4), and the Bray-Cutis dissimilarity PCoA plot shows a very clear separation between the samples from Java (Fig 3B; open circles) and those from East Nusa Tengarra (closed circles). Similar separation between the two datasets are also observed for the other β-diversity metrics (S2 Fig). Also, some differences in α-diversity between the two dataset are eminent (Table 4).

**Fig 4:**
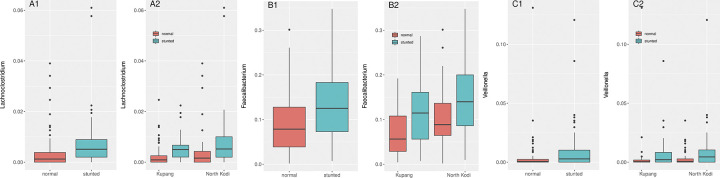
Boxplots of the 3 taxa at genus level that were significantly different (Kruskal-Wallis analysis, with Benjamini-Hochberg FDR correction) between non-stunted (N; red) and stunted (S; green) children in the full dataset (panels 1) and separated out by sampling site (panels 2). A1 + A2. *Lachnoclostridium*; B1 + B2. *Faecalibacterium*; C1 + C2. *Veillonella*.

The total number of taxa at the genus-level in the full dataset was 412. After filtering for those that were present in at least 20% of the samples, 125 genus-level taxa remained, indicating that the majority of genera was present in only a few children. Of the 125 taxa remaining, 52 (41.6%) were significantly (*q* < 0.05) different between sampling sites using the non-parametric Kruskal-Wallis (KW) analysis, possibly influenced by previous observed differences between rural and urban microbiota composition [35,36], observed already as early as 2001 [37]. We did not investigate this further, as we were interested in the differences correlated with stunting. Taking the full dataset (all 200 children), KW analysis indicated 30 taxa to be significantly different between stunted children and non-stunted children (*q* < 0.05). However, after segregating the data into the rural and urban sampling sites, only 3 taxa were consistently different between stunted children and non-stunted children at both sampling sites (Fig 4): *Lachnoclostridium* (*q* = 3.1 * 10^−6^), *Faecalibacterium* (*q* = 3.1 * 10^−4^), and *Veillonella* (*q* = 6.7 * 10^−4^).

In our previous study, *Prevotella 9* was the most prevalent taxon, with on average 26.3% relative abundance (RA) [27], and it was lower in stunted children on Java (23.5% for stunted children vs. 30.5% for non-stunted children; *q* = 0.058). In the current study however, the RA of *Prevotella 9* was much lower (on average 9%), and opposed to our previous results was higher in the stunted children than in the children with normal nutritional status (9.95% for stunted children vs. 8.04% for non-stunted children; *q* = 0.36). *Prevotella* has previously been linked with non-Western subjects [38]. However, it has also been linked to ingestion of (plant) carbohydrates [39]. How *Prevotella* relates to stunting exactly remains to be seen given the disparity in results from our two studies.

In the current study *Faecalibacterium* was the taxon with average highest RA (11.1%; 13.3% for stunted children vs. 8.8% for non-stunted children; *q* = 3.1 * 10^−4^; Fig 4B1). *Faecalibacterium* was slightly higher in the children from North Kodi compared to those from Kupang (Fig 4B2). Interestingly, *Faecalibacterium* is considered to be a biomarker for health, with associations with numerous diseases and disorders, such as IBD (particularly Crohn’s disease) [40] and obesity [41]. It is therefore counterintuitive to find higher RA of *Faecalibacterium* in stunted children.

*Lachnoclostridium* has previously been associated with autism spectrum disorder (ASD) in children [42], and is used as a novel marker for colorectal cancer in adults [43]. It also has been shown to be involved in production of secondary bile acids in critically ill children [44]. *Veillonella* are opportunistic pathogenic bacteria. The overgrowth of *Veillonella* is associated with many diseases, including oral diseases, endocarditis, and ulcerative colitis, and an overabundance of *Veillonella* promotes intestinal inflammation [45]. *Veillonella* has also been shown to be modulated in ASD, but not consistently, with some studiesshowing increased *Veillonella* and others decreased *Veillonella* in ASD [42].

**Fig 5:**
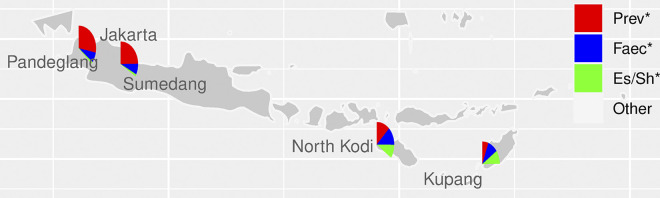
Distribution of the taxa *Prevotella 9* (Prev, red), *Faecalibacterium* (Faec, blue), *Escherichia/ Shigella* (Es-Sh, green), and the summed other taxa (Other, transparent) at the different sampling sites from the current study and our previous study [27]. Of the 133 taxa in the combined dataset, 87 were signficiantly different between the two regions (*q* < 0.05). for the three most prevalent taxa in this figure the *q*-values are *Prevotella 9* 6.0 * 10^−22^, *Faecalibacterium*: 5.8 * 10^−4^, *Escherichia/ Shigella*: 3.1 * 10^−22^. Data from the Java study is from [27].

The second highest taxon in the current study was *Escherichia/Shigella*. It was highly prevalent, 9.7% in stunted children and 9.3% in non-stunted children, but not significantly different between the two groups (*q* = 0.71). Strikingly, in children in East Nusa Tenggara (ENT) (9.5%) this taxon was approximately 7-fold higher than in children on Java (1.4%; the green color can hardly be observed in Fig 5 for the Java samples). This may be related also to observed differences in diarrheal prevalence in these provinces: diarrhea prevalence in ENT, Banten and West Java is 9.1%, 8.4%, 5.4%, respectively [11]. The same data from the Indonesian ministry of health also revealed that access to proper drinking water varies between these provinces: in ENT, West Java, and Banten it is 46.6%, 67.6%, and 72%, respectively. Moreover, proper sanitation in ENT is also lower than on Java: with 62.9% in ENT versus 82.1% and 75.2% in Banten and West Java, respectively [11]. Differences in culture, education, infrastructure facilities are also quite diverse between Java and Nusa Tenggara islands, and these may contribute to the gut microbiota profile of the young children.

The three taxa that were significantly different between the two groups of children in East Nusa Tengarra were not significantly different between the two groups of children on Java, and vice versa. Strikingly, *Veillonella* was only present in 1 child in the Java-samples. Although there was a difference in average age of the two studies (average 46.8 months within Java-study, average 39.7 months within the ENT-study), and although age does influence gut microbiota composition as reviewed by Selma-Royo *et al*. [46], none of the three taxa different between stunted children and non-stunted children were correlated with age, not in the current study, nor if both datasets were combined.

Other studies have looked at the microbiota differences between stunted and normal children. In another region in Indonesia, on the northern tip of Sumatra, the gut microbiome profle of stunted children (n = 21) showed enriched genera such as *Blautia*, *Dorea*, *Collinsella*, *Streptococcus*, *Clostridium sensu stricto 13*, *Asteroleplasma* and *Anaerostipes*. Meanwhile, compared to the normal children (n = 21), there was a depletion of *Prevotella*, *Lactococcus*, *Butyrivibrio*, *Muribaculaceae*, *Alloprevotella*, *Akkermansia*, *Enterococcus*, *Terrisporobacter* and *Turicibacter* [47]. In a small longitudinal analysis of the gut microbiota in persistently stunted young children in South India (n = 10 children in each group), the RA of the Bacteroidetes phylum was higher in stunted children compared to that of control children at 12 months of age but otherwise did not find significant differences in diversity indices between cases and controls. The microbiota of control children was enriched in *Bifidobacterium* and *Lactobacillus* species, whereas that of stunted children was enriched in inflammogenic taxa including those in the Desulfovibrio genus and Campylobacterales order [48]. In another study on South-Indian children [49], stunted children (n = 16) showed enrichment for bacterial genera including *Prevotella 7*, *Prevotella 9* and *Sutterella*. They also showed depletion of Clostridiaceae 1 family, *Bifidobacterium*, *Intestinibacter* and *Fusicatenibacter* genera compared to non-stunted children (n = 25). In 75 infants aged 5–12 months living in rural areas of Peru that were followed for 6 months, the RA of several bacterial taxa were found to increase over time in children that became stunted (n = 16) but not in non-stunted children (n = 49). These included Ruminococcus 1, Ruminococcus 2, Ruminococcaceae UCG 014, Lachnospiraceae, Clostridiaceae 1, and Collinsella. Providencia decreased over time in children that became stunted [50]. In the only other large study, in 335 children from rural Zimbabwe from 1–18 months of age, the authors found, using whole metagenomic sequencing, that taxonomic microbiome features are poorly predictive of child growth, however functional metagenomic features, particularly B-vitamin and nucleotide biosynthesis pathways, moderately predict both growth and growth velocity. The prevalence of stunting varied from 18–34% across study time-points [51].

A recent review describes decreased microbial diversity and changes in specific bacterial taxa in malnourished children. These alterations have been linked with compromised gut barrier function, immune dysregulation, and systemic spread of the gut microbiota, but changes in composition between different studies were not consistent [52].

### Correlations between gut microbiota and anthropometric measures

**Fig 6:**
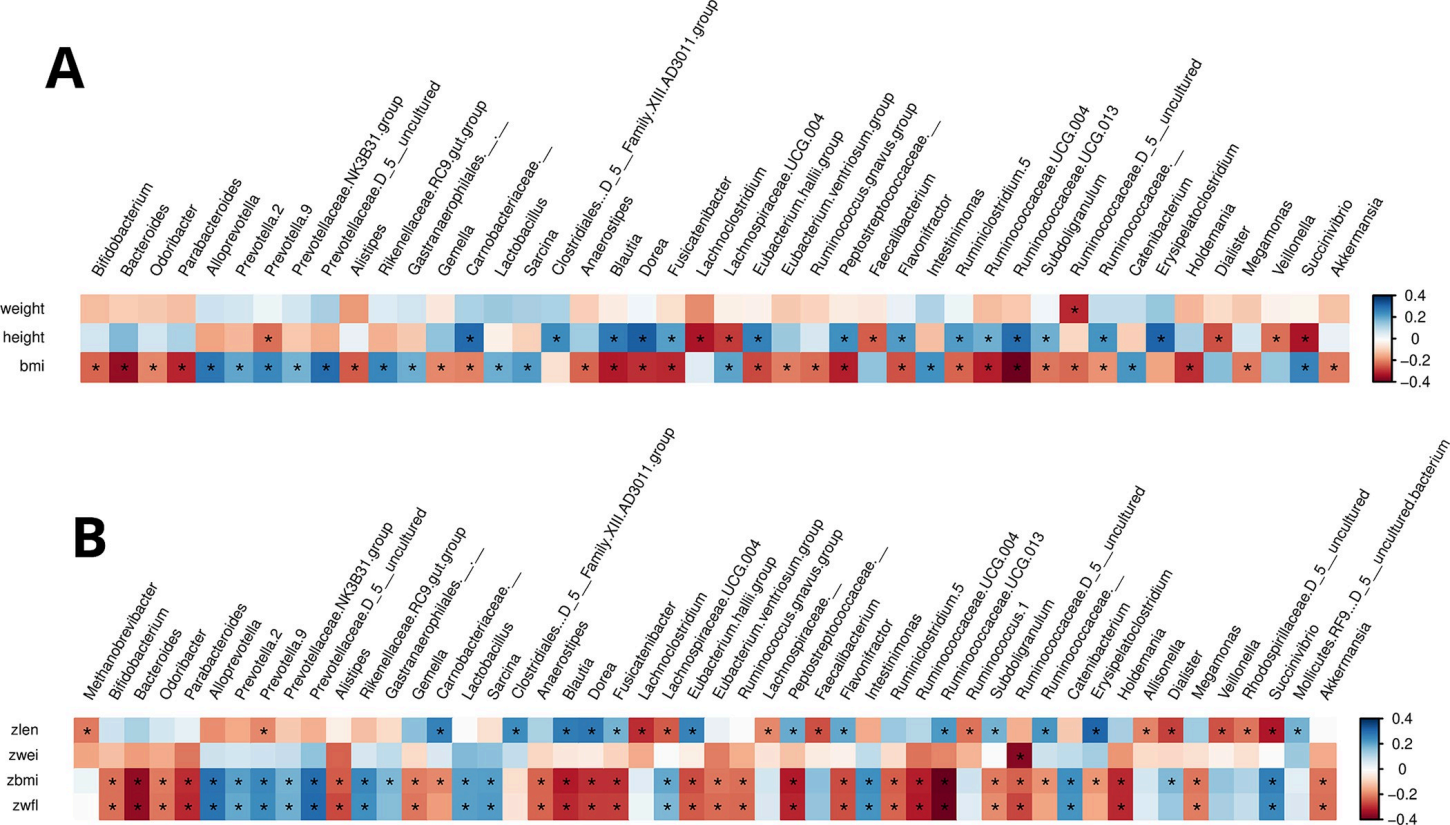
Heatmap of the taxa at genus level that were different (*q* < 0.05) when correlated by Spearman correlation analysis to the anthropometric indices (A) and the corresponding z-scores (B). Cells are colored by rho (correlation coefficient): Blue: Positive correlation; red: Negative correlation. Significance (q < 0.05) is indicated by *. Abbreviations: Bmi: Body-mass index; zlen: Z-score for length-for-age; zwei: Z-scor for weight-for-age; zbmi: Z-score for bmi-for-age; zwfl: Z-score for weight-for-length.

Fig 6A shows the taxa that are significantly (q < 0.05) correlated (Spearman) with height, weight and bmi, and Fig 6B with the corresponding z-scores. The cells are colored by the correlation coefficient (rho). As expected from the significantly higher RA of *Faecalibacterium*, *Lachnoclostridium* and *Veillonella*, these are negatively correlated with height and zlen.

As expected there is significant overlap between the taxa in Fig 6A and the metrics derived from the anthropometric indices in Fig 6B, although there are a few differences. Most taxa are correlated to bmi and zbmi (and the similar zwfl). Only an uncharacterized genus in the Ruminococcacea family correlates negatively to weight and zwei. Besides *Faecalibacterium*, *Lachnoclostridium* and *Veillonella*, there are 5 (Fig 6A) and 9 (Fig 6B) other taxa that are negatively correlated to height and zlen, respectively, while there are 14 (Fig 6A) and 13 (Fig 6B) taxa that are positively correlated to height and zlen, respectively.

### Correlations between gut microbiota and sanitary parameters

**Fig 7:**
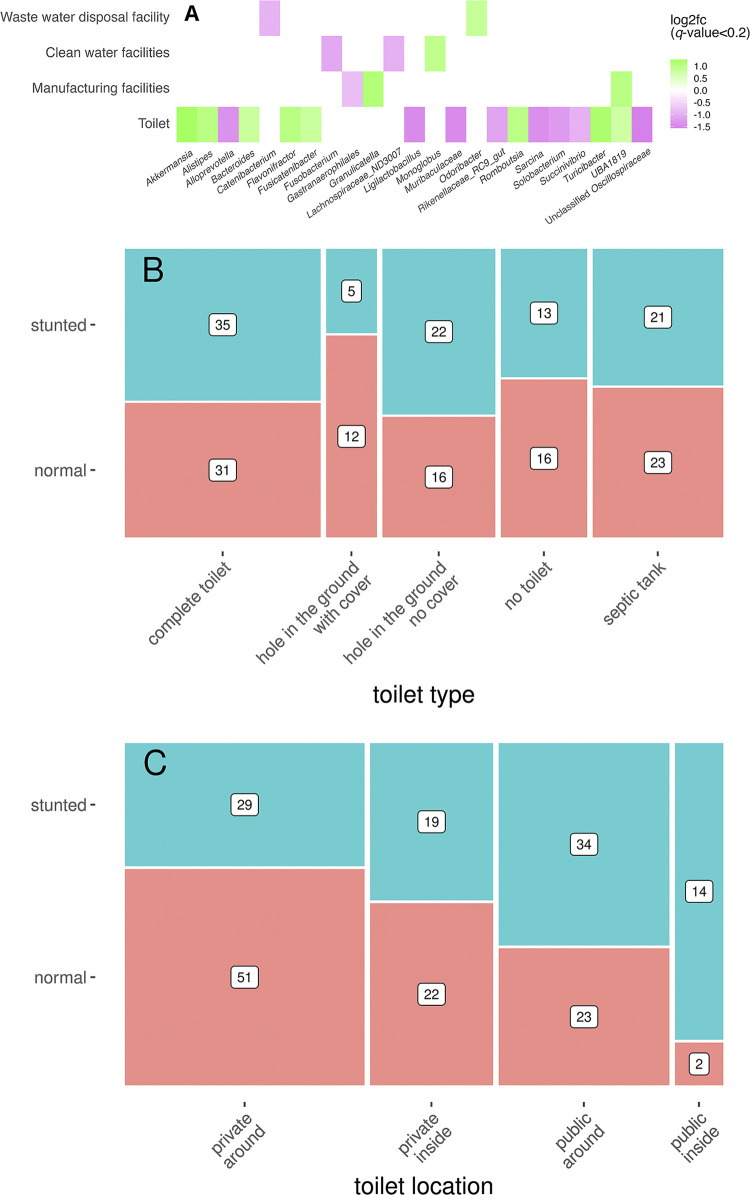
A) Heatmap of the significant microbial taxa correlating by Spearman correlation analysis (*q* < 0.05) with the sanitary parameters ‘Waste water disposal facility’, ‘Clean water facilities’, ‘Trash bins’, and ‘Toilet type’. B and C) Mosaic plots showing the relative frequency of observations within each of the two groups of children (stunted; blue and normal; orange) with toilet type (B, *p* = 0.36) and (public) toilet and location (C, *p* = 0.0006). The relative percentage are shown within each group.

Several taxa correlated with sanitary parameters (Fig 7A). Type of toilet was correlated to several taxa (Fig 7A), but not significantly different between the two groups (Fig 7B). However, whether it was public or private, and located inside the accommodation or elsewhere were important discriminators between the two groups (Table 2, Fig 7C).

Interestingly, the three taxa that were significantly different between the two groups, *Lachnoclostridium*, *Faecalibacterium* and *Veillonella*, were not among the taxa that correlated with the hygienic parameter ‘toilet type’ (Fig 7). Instead, *Akkermansia*, a taxon generally considered to be correlated with health, did show a correlation with toilet type (*q* = 0.0087). How this relates to stunting remains to be seen, as it was not correlated to being stunted or not (*q* = 0.26). Rinanda et al. [47] noticed that water contaminants such as *Aeromonas*, *Stappiaceae*, and *Synechococcus* were higher in stunted children compared to normal children. In our study, an overall increase in the *Escherichia*/*Shigella* taxon, usually correlated with hygiene, was observed (not specifically in stunted children), as well as a specific increase in stunted children in the phylum Cyanobacteria, of which the natural habitat is considered to be water environments.

### Correlations between gut microbiota and microbial metabolites

Fecal SCFA concentrations were measured as well. Although we acknowledge that these concentrations cannot be interpreted into SCFA production by the microbiota, it is interesting to observe the correlations between the taxa and the presence of these microbial metabolites in feces. It is difficult to convert SCFA concentrations to SCFA production, because first of all, if concentrations between the groups is the same, but fecal output (which was not recorded) differs, then the absolute amount of excreted SCFA differs too (despite the same concentrations). Moreover, an increased fecal excretion can be caused by a lower intestinal absorption, and thus does not necessarily mean increased production. Despite this, all three SCFA concentrations were lower in stunted children, compared to non-stunted children, although only significant for acetate and propionate (Table 5). As a consequence, also total SCFA was also lower in stunted children than in children with normal zlen (p = 0.011). If this could be translated to lower SCFA production, there is less energy-extraction from the diet by the gut microbiota in the stunted children. Also, since SCFA have been shown to be involved in gut barrier function [14,17], this barrier may be negatively affected in the stunted children. Alterations in the gut microbiota have been linked with compromised gut barrier function, immune dysregulation, and systemic spread of the gut microbiota, as discussed by Jones et al. [52] and highlighted above. However, SCFA production as not one of the metabolic pathways associated with growth or growth velocity in the only metagenomic study in children living in rural Zambia [51].

**Table 5:** SCFA concentrations (mmol/g wet weight) in fecal samples.

	stunted	normal	*p*-value
**acetate**	27.44 ± 7.83	31.15 ± 9.15	0.003 *
**propionate**	12.15 ± 6.05	14.4 ± 8.50	0.035 *
**butyrate**	9.57 ± 3.94	9.83 ± 4.47	0.67
**total SCFA**	49.17 ± 13.98	55.40 ± 19.00	0.011 *

**Fig 8:**
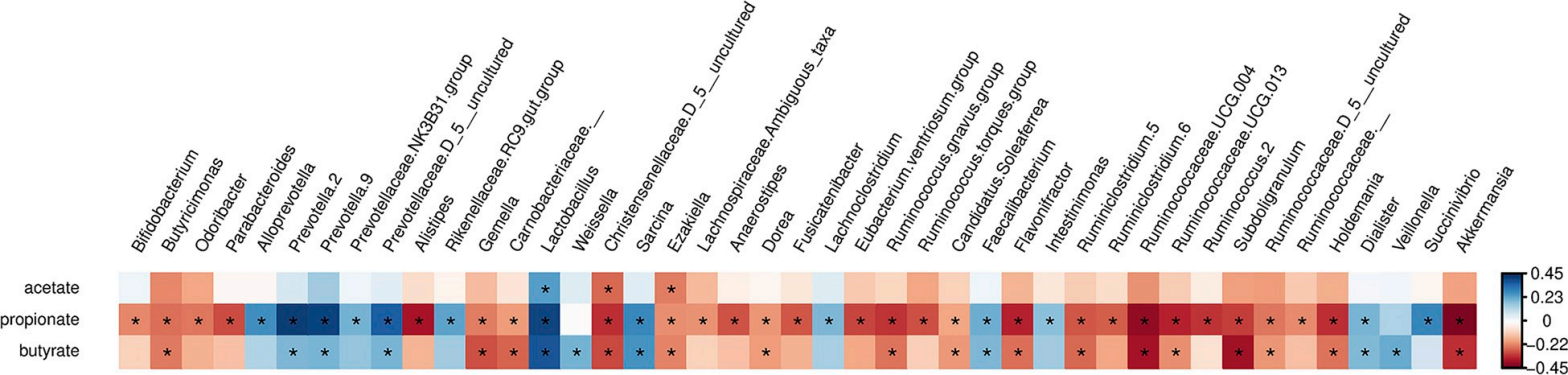
Heatmap of the taxa at genus level that were different when correlated by Spearman correlation analysis (*q* < 0.05) to the fecal concentrations of the SCFAs acetate, propionate and butyrate. Cells are colored by rho (correlation coefficient): Blue: Positive correlation; red: Negative correlation.

Fig 8 shows the Spearman correlation coefficients for the taxa that significantly correlated to the fecal SCFA concentrations. Most taxa correlated to propionate, either positively or negatively, even if those taxa are not considered to be directly involved in propionate production, such as *Faecalibacterium*, which produces butyrate. None of the other studies on stunting or growth have looked at SCFA in feces.

The strength of this study is the inclusion of 100 children in each group. Most previous studies have only looked at a limited numbers of stunted children [47–51]. However, the study also has limitations. All taxa currently at most correlate to being stunted; no cause and effect relationship has been established. Also, as is clear from our own two studies in Indonesia (on Java and ENT), as well as the study by Rinanda et al. [47] on Sumatra, the composition of the gut microbiota, even within one country, is geography specific, which is corroborated by the studies in Zimbabwe, India and Peru [47–51]. Hence no general conclusions can be drawn. On top of that, differences in library preparation, sequencing primers, PCR cycles or bioinformatics tools may lead to different results [53]. Although for our study on Java and ENT different libraries were prepared (in fact even within those studies more than 1 library was necessary due to the number of samples), all other tools were identical. Based on our positive control and mock control there were no differences between the sequence runs for those two geographical location, and no other technical difference were present (primers, number of PCR-cycles, etc.). As such, the difference in composition between the gut microbiota on Java and ENT is believed to be primarily due to geographical location, and influenced by differences in diet, hygienic status and environmental conditions.

## Conclusion

In conclusion, the relationship between gut microbiota and stunting is complex, and is influenced by many confounding factors. In the current study three taxa were consistently different between stunted children and non-stunted children at both sampling sites in East Nusa Tenggara. However, these were different taxa compared to our study on Java, even though this was in the same country, partly as a consequence of the different composition of the samples from ENT altogether. Differences in diet, cultural habits, but certainly also infrastructure facilities such as clean water and sanitation are expected to contribute to the differences between our studies on Java and ENT, as we show here that these sanitary parameters are different between the two groups, and correlate to several taxa. Based on recent results from Robertson at el. [51] on whole metagenome sequencing, it may not be sufficient to indicate differences in taxa, but important biochemical pathways may have to be elucidated, including those for SCFA production, which link to barrier function and immune modulation. Further, it remains to be seen whether the taxa that positively correlated with zlen are involved in stunting, and whether increasing the ones that are negatively correlated with zlen might correct some of the growth retardation. In the meantime, we are performing a food intervention on these children to investigate this.

## Supporting information

S1 Fig Principal coordinate analysis plot of A) unweighted UniFrac, B) weighted UniFrac, C) Jaccard similarity for non-stunted (red) and stunted (blue) children of the current study in East Nusa Tenggara.
(TIF)

S2 Fig Principal coordinate analysis plot of A) unweighted UniFrac, B) weighted UniFrac, C) Jaccard similarity for the non-stunted (red) and stunted (blue) children of the current study in East Nusa Tenggara (full circles) and our previous study on Java (open circles).
(TIF)

## Abbreviations

16S rRNA: small subunit ribosomal RNA
ASD: autism spectrum disorder
ASVs: Amplified Sequence Variants
BMI: body-mass index
ENT: East Nusa Tenggara
FDR: false discovery rate
GC-MS: gas-chromatography mass-spectometry
KW: Kruskal-Wallis
OTU: operational taxonomic unit
PBS: Phosphate buffered saline
PCoA: principal coordinate analysis
PD: phylogenetic diversity
PERMANOVA: Permutational multivariate analysis of variance
QIIME: Quantitative Insights Into Microbial Ecology
RA: relative abundance
SCFAs: short-chain fatty acids
UNICEF: United Nations Children’s Fund
UniFrac: unique fraction
V3-V4: variable regions 3 and 4
WHO: World Health Organisation
zbmi: bmi-for-age
zlen: height/weight for length
zwei: weight-for-age
zwfl: weight-for-length
