# Supplementary figures and images for: Gut microbiota differences in stunted and normal-lenght children aged 36–45 months in East Nusa Tenggara, Indonesia
Source: PLoS One. 2024 Mar 29;19(3):e0299349. doi: 10.1371/journal.pone.0299349 (PMC10980242; doi:10.1371/journal.pone.0299349)

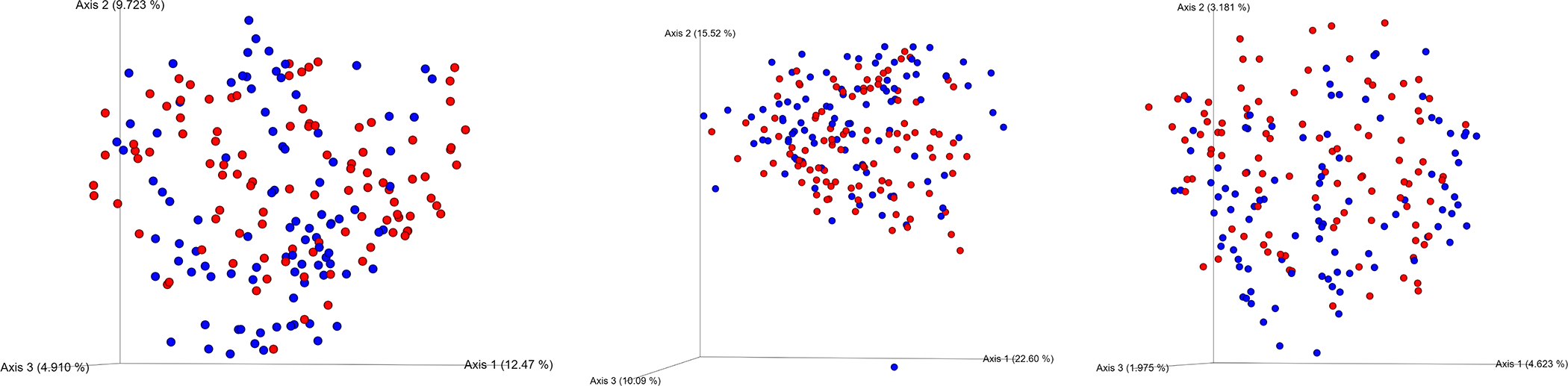

Supplement: S1 Fig — Principal coordinate analysis plot of A) unweighted UniFrac, B) weighted UniFrac, C) Jaccard similarity for non-stunted (red) and stunted (blue) children of the current study in East Nusa Tenggara. (TIF) [file pone.0299349.s001.tif]

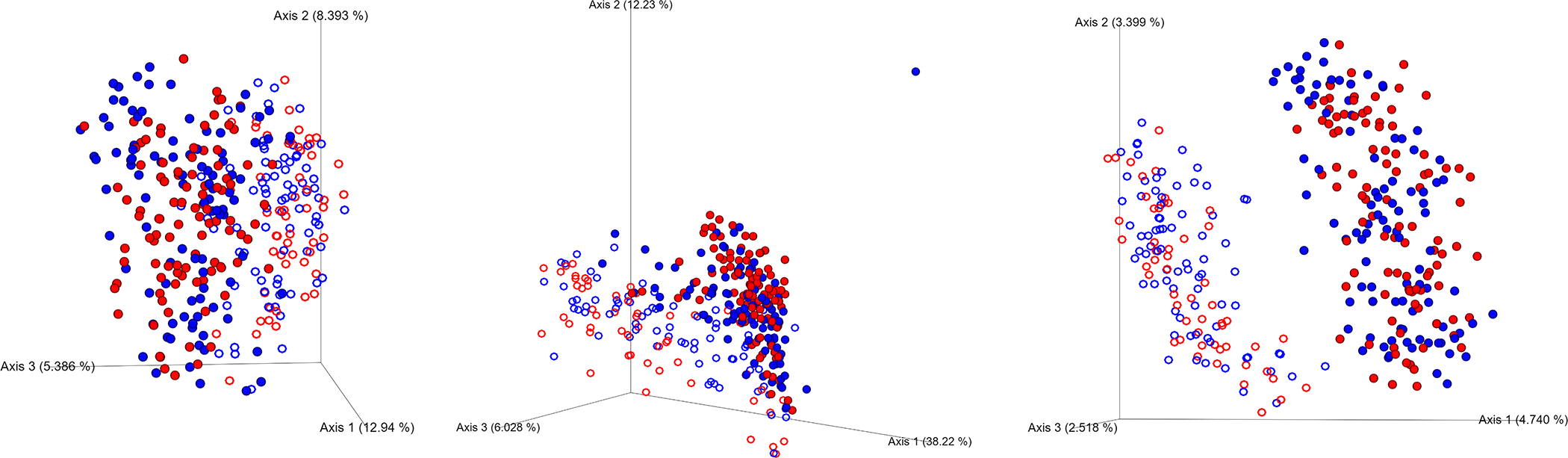

Supplement: S2 Fig — Principal coordinate analysis plot of A) unweighted UniFrac, B) weighted UniFrac, C) Jaccard similarity for the non-stunted (red) and stunted (blue) children of the current study in East Nusa Tenggara (full circles) and our previous study on Java (open circles). (TIF) [file pone.0299349.s002.tif]
